# Prevalence of serological response to *Borrelia burgdorferi* in farmers from eastern and central Poland

**DOI:** 10.1007/s10096-016-2813-7

**Published:** 2016-10-31

**Authors:** V. Zając, J. Pinkas, A. Wójcik-Fatla, J. Dutkiewicz, A. Owoc, I. Bojar

**Affiliations:** 1 Department of Health Biohazards and Parasitology, Institute of Rural Health, Jaczewskiego 2, 20-090 Lublin, Poland; 2 School of Public Health, Centre of Postgraduate Medical Education, Kleczewska 61/63, 01-826 Warsaw, Poland; 3 Center for Public Health and Health Promotion, Institute of Rural Health, Jaczewskiego 2, 20-090 Lublin, Poland; 4 Department for Women’s Health, Institute of Rural Health, Jaczewskiego 2, 20-090 Lublin, Poland

## Abstract

Lyme borreliosis (Lyme disease) caused by the *Borrelia burgdorferi* sensu lato spirochete is the most common tick-borne infection manifested by a wide spectrum of clinical symptoms. In Poland, the preventive health care does not comprise individual farmers as it is practiced in foresters. The objective of this study was to evaluate the exposure of Polish farmers to infection with *B. burgdorferi*, based on serological screening test and epidemiological investigation. A total of 3,597 farmers were examined for the presence of *B. burgdorferi* antibodies, as well as interviewed regarding exposure to ticks and prophylaxis of tick-borne diseases. The prevalence varied between 18.2 and 50.7 % suggesting a focal occurrence of borreliosis. A significant increase in the frequency of positive reactions in the oldest age ranges was observed, equaling 30.9 % in the range of 60–69 years and 53.6 % in the range of 80–91 years. The prevalence of the anti-*B. burgdorferi* antibodies of IgG class (14.7 %) was similar to that of IgM class (16.0 %). Seroreactivity to *B. burgdorferi* antigen was significantly higher in the group of farmers exposed to repeated tick bites. Significant relationships were also found between some other risk factors and occurrence of seropositive reactions to *B. burgdorferi*. To the best of our knowledge, this is the first study concerning *s*eroprevalence to *B. burgdorferi* carried out on such a large group of farmers. Results indicate a high risk of *B. burgdorferi* infection among Polish farmers and associations between some risk factors and the presence of seropositive reactions.

## Introduction

Lyme borreliosis (Lyme disease) caused by the spirochete *Borrelia burgdorferi* sensu lato is the most common tick-borne infection, both in Europe and the United States, which is manifested by a wide spectrum of clinical symptoms. The most common clinical manifestation is erythema migrans, which eventually resolves, even without antibiotic treatment. However, the infecting pathogen can spread to other tissues and organs, causing severe manifestations involving skin, nervous system, joints, or heart. The incidence of this disease is increasing in many countries [1, 2]. Neurologic involvement occurs in 10–15 % of untreated *B. burgdorferi* infections [3, 4]. At present, Lyme borreliosis is the most frequent occupational disease recorded in Poland. In 2014, 2,351 cases of occupational diseases were registered in Poland, including 660 cases of infectious and parasitic diseases, among which Lyme disease constituted 82.3 % of the total [5].

To assess the risk of the *B. burgdorferi* infection, mainly indirect epidemiological methods have been used, such as seroprevalence surveys or estimation of the prevalence of infected ticks. Until recently, the other risk factors, including type of occupation or contact with certain animal species, have not been well documented [6].

An occupational risk to *B. burgdorferi* infection among farmers was demonstrated during recent decades by a few authors [6–12]. The studies conducted in Poland show that the risk of *B. burgdorferi* infection in farmers is comparable to that in forestry workers, and in some cases, it is even higher [7, 8, 11]. The high risk of exposure to infection with *B. burgdorferi* and other tick-borne pathogens creates the necessity to elaborate effective prevention measures in this occupational group, the more so, as no effective vaccine against Lyme borreliosis is currently available. In Poland, individual farmers are not covered by preventive health care related to Lyme borreliosis, in contrast to foresters [13].

Taking into account the above-mentioned data, the objective of this study was to evaluate the exposure of farmers from three regions of Poland to infection with *B. burgdorferi* spirochetes, based on serological screening test and questionnaire examination.

## Materials and method

### Examined population

**Fig. 1:**
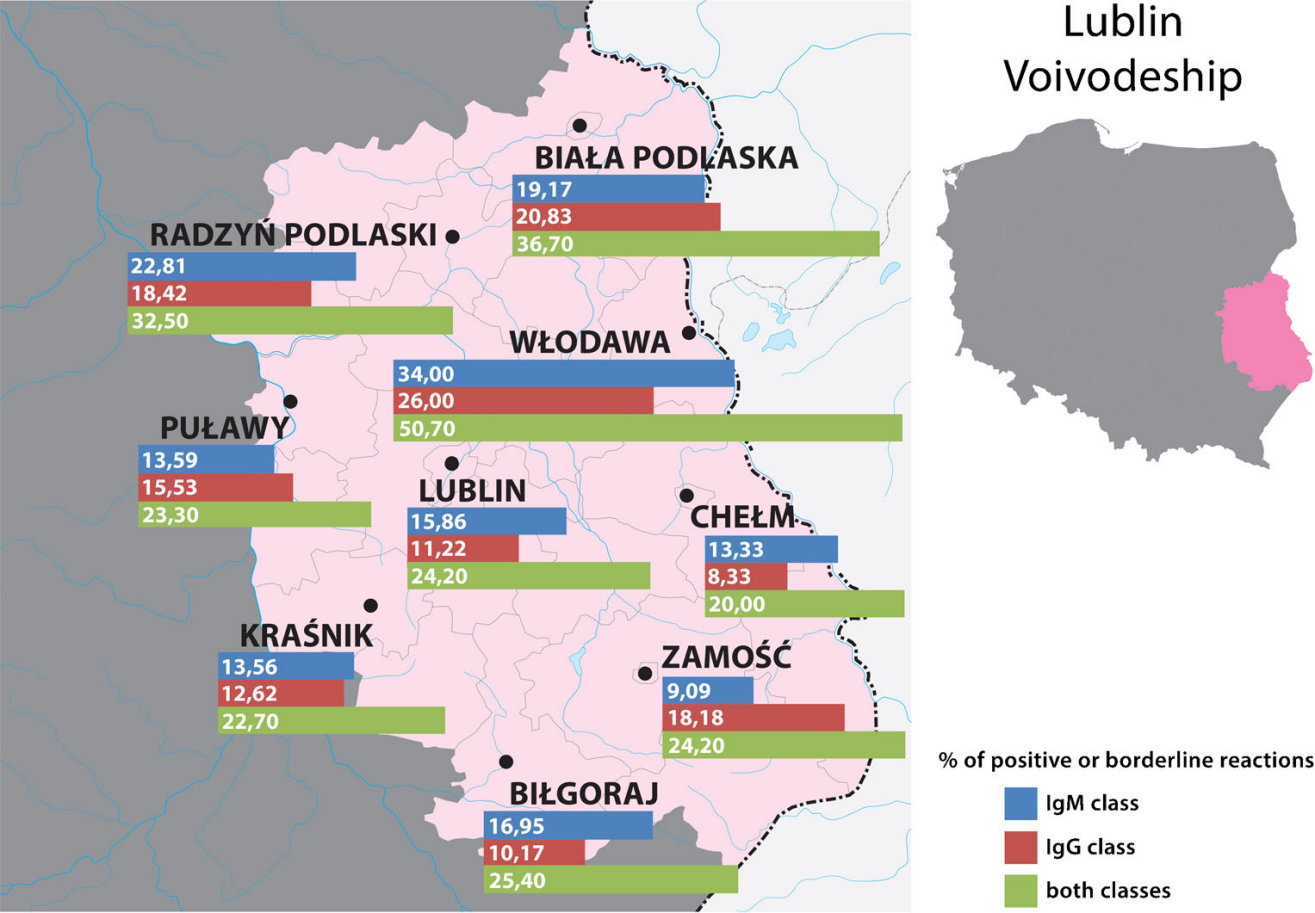

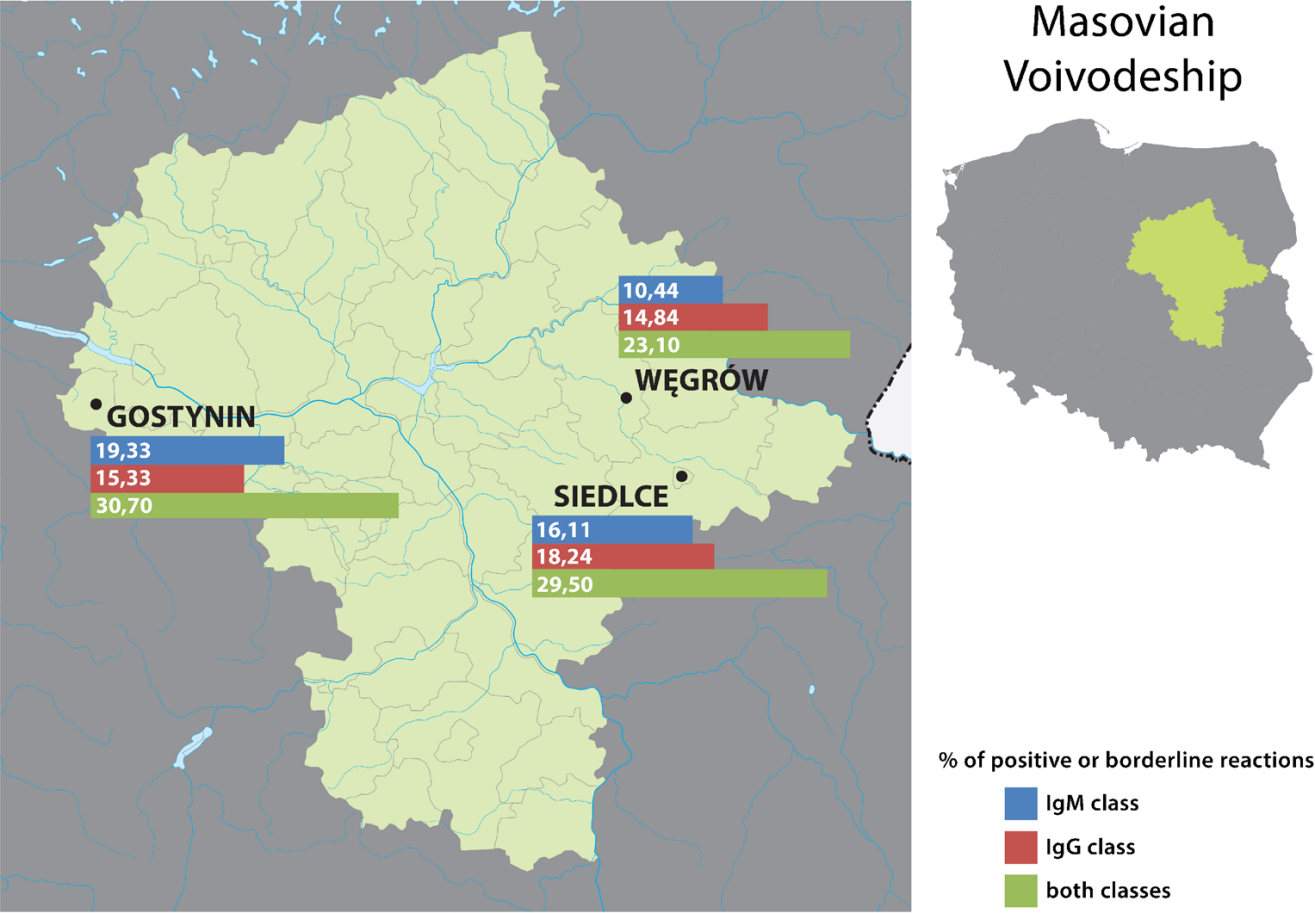

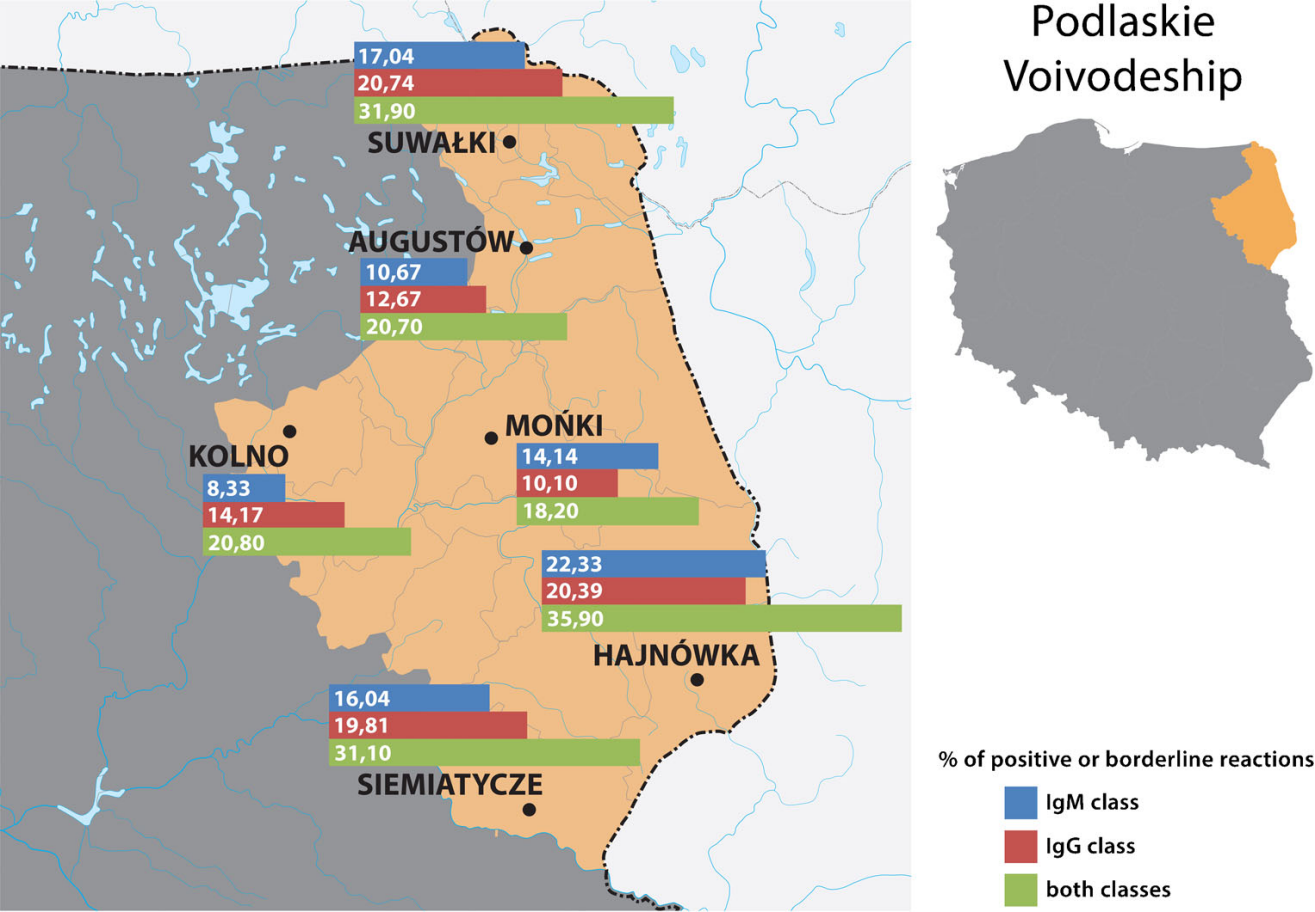
Localities in which the farmers lived who were examined in the study and prevalence of serological response to *B. burgdorferi*

A total of 3,597 farmers living in 18 localities on the area of three voivodeships (provinces): Lubelskie, Mazovian and Podlaskie (Fig. 1), were examined from September 2015 until February 2016. There were two criteria for inclusion in the study: (1) performance of the farmer’s occupation, and (2) coverage by the Polish Agricultural Social Insurance Fund (KRUS). The sample of examined farmers comprised 1,469 males and 2,128 females, and the mean age amounted to 51.3 ± 11.4 years. Two of them had a clinical form of Lyme borreliosis diagnosed by a doctor during performance of the study, and another two persons reported receiving an antibiotic therapy because of an infectious disease. Moreover, two persons reported autoimmune diseases (rheumatoid arthritis and myasthenia gravis). All the examined population was interviewed regarding exposure to ticks and prophylaxis of diseases transmitted by ticks with the use of an original questionnaire elaborated in the Institute of Rural Health. Blood samples were collected by the puncture of a forearm vein for later serological examination with *B. burgdorferi* antigen. Each of the subjects signed informed consent approved by the Bioethical Commission of the Institute of Rural Health (Permission No. 08/2015).

### Serological examination for the presence of anti- *B. burgdorferi* antibodies

Sera of farmers from the study area were examined for the presence of specific anti-IgM and anti-IgG antibodies to *B. burgdorferi* sensu lato (s.l.) with the use of the commercial ELISA test (*B. burgdorferi* recombinant IgM and *B. burgdorferi* recombinant IgG, Biomedica Medizinprodukte GmbH and Co. KG, Vienna, Austria) according to the manufacturer’s instruction. In both serological kits, recombinant proteins of *B. burgdorferi* s.l. were used as an antigen (p18, p100 and VlsE for IgG kit and OspC, p41/l and VlsE for IgM kit). The results were calculated in BBU/ml (Biomedica Borrelia Units); results equal to 11 BBU/ml and above were considered as positive, those between 9 and 10 BBU/ml were considered as borderline, and those below 9 BBU/ml were considered as negative. The sensitivity and specificity of kits were 100 and 96 %, respectively.

### Statistical analysis

The associations between prevalence of seropositive reactions depending on locality as well as on age and gender of examined farmers were analyzed by χ^2^ test and Student’s t-test, using the STATISTICA v. 6.0 package (Statsoft, Tulsa, OK, USA). The significance of the associations between prevalence of seropositive reactions and questionnaire data was assessed by odds ratio calculation using MedCalc® software [14]. The value of *P* < 0.05 was considered as significant.

## Results

Table 1 presents the prevalence of seropositive reactions to *B. burgdorferi* in the farmers inhabiting eastern and central Poland, depending on locality. The prevalence varied between 18.2 and 50.7 %, depending on locality, and this variation proved to be highly significant (χ^2^ = 59.599; *P* < 0.000001) (Table 1, Fig. 1), suggesting a focal occurrence of borreliosis. However, the mean prevalence calculated for each of three voivodeships was within the narrow range of 26.2–28.0 % (mean 26.8 %), but this variation was not significant (χ^2^ = 0.464; *P* = 0.793).

**Table 1:** Prevalence of antibodies against *Borrelia burgdorferi* in farmers from eastern and central Poland depending on geographic location

Voivodeship	Locality	Number of farmers examined	Reactions with antibodies belonging to IgM class				Reactions with antibodies belonging to IgG class				Positive or borderline reactions in one or both classes	
			Positive		Borderline		Positive		Borderline			
			Number	Percent %	Number	Percent %	Number	Percent %	Number	Percent %	Number	Percent %
Lublin	Lublin	1141	131	11.5	50	4.5	118	10.3	10	0.9	276	24.2
	Biała Podlaska	120	17	14.2	6	5.0	24	20.0	1	0.8	44	36.7
	Biłgoraj	59	7	11.9	3	5.1	6	10.2	0	0.0	15	25.4
	Chełm	120	10	8.3	6	5.0	7	5.8	3	2.5	24	20.0
	Kraśnik	317	27	8.5	16	5.0	36	11.4	4	1.3	72	22.7
	Puławy	103	10	9.7	4	3.9	14	14.0	2	1.9	24	23.3
	Radzyń Podlaski	114	22	19.3	4	3.5	19	16.7	2	1.8	37	32.5
	Włodawa	150	38	25.3	13	8.7	36	24.0	3	2.0	76	50.7
	Zamość	99	8	8.1	1	1.0	16	16.2	2	2.0	24	24.2
Total		2223	270	12.2	103	4.6	276	12.4	27	1.2	592	26.6
Masovian	Gostynin	150	20	13.3	9	6.0	22	14.7	1	0.7	46	30.7
	Siedlce	329	34	10.3	19	5.8	55	16.7	5	1.5	97	29.5
	Węgrów	182	16	8.8	3	1.7	25	13.7	2	1.1	42	23.1
Total		661	70	10.6	31	4.7	102	15.4	8	1.2	185	28.0
Podlaskie	Hajnówka	103	21	20.4	2	1.9	21	20.4	0	0.0	37	35.9
	Augustów	150	9	6.0	7	4.7	19	12.7	0	0.0	31	20.7
	Kolno	120	8	6.7	2	1.7	17	14.2	0	0.0	25	20.8
	Mońki	99	7	7.1	7	7.1	10	10.2	0	0.0	18	18.2
	Siemiatycze	106	11	10.4	6	5.7	21	19.8	0	0.0	33	31.1
	Suwałki	135	17	12.6	6	4.4	25	18.5	3	2.2	43	31.9
Total		713	73	10.2	30	4.2	113	15.6	3	0.4	187	26.2
Total in all voivodeships		3597	413	11.5	164	4.6	491	13.7	38	1.1	964	26.8

Variation depending on locality, assessed by *χ* ^2^ test: χ^2^ = 59.599, *P* < 0.000001, variation highly significant

**Fig. 2:**
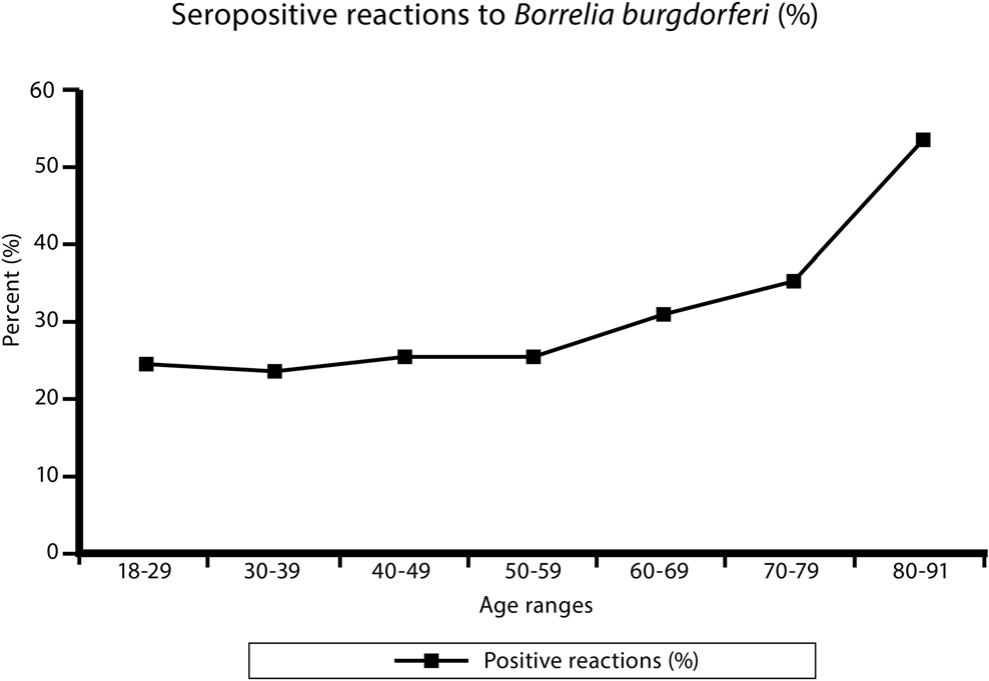
Prevalence of anti-*B. burgdorferi* antibodies depending on age of the tested individuals

Table 2 presents the prevalence of seropositive reactions to *Borrelia burgdorferi* in the farmers, depending on gender and age. No significant difference could be found between the groups of female and male farmers (26.3 % vs. 27.5 %; *P* = 0.432). In contrast, there was noted a significant variation in the frequency of seropositive reactions depending on age (χ^2^ = 18.109; *P* = 0.006). Figure 2 clearly shows that this variation was associated with an increase of the frequency of positive reactions in the oldest age ranges, from 30.9 % in the range 60–69 years, to 53.6 % in the range 80–91 years, distinctly higher compared to mean frequency of 26.8 % in the total examined population (Fig. 2).

**Table 2:** Prevalence of antibodies against *Borrelia burgdorferi* in farmers from eastern and central Poland depending on the gender and age

Parameter	Number of examined farmers	Reactions with antibodies belonging to IgM class				Reactions with antibodies belonging to IgG class				Positive or borderline in one or both classes	
		Positive		Borderline		Positive		Borderline			
		Number	Percent %	Number	Percent %	Number	Percent %	Number	Percent %	Number	Percent %
Gender											
Females	2128	257	12.1	110	5.2	248	11.7	21	1.0	560	26.3
Males	1469	156	10.6	54	3.7	243	16.5	17	1.2	404	27.5
Age (in years)											
18–29	127	17	13.4	9	7.1	8	6.3	1	0.8	31	24.4
30–39	440	64	14.6	24	5.5	30	6.8	5	1.1	106	24.1
40–49	1001	125	12.5	52	5.2	108	10.8	10	1.0	253	25.3
50–59	1268	131	10.3	49	3.9	180	14.2	9	0.7	326	25.7
60–69	577	60	10.4	25	4.3	111	19.2	10	1.7	178	30.9
70–79	156	12	7.7	4	2,6	44	28.2	2	1.3	55	35.3
80–91	28	4	14.3	1	3.6	10	35.7	1	3.6	15	53.6
Total	3597	413	11.5	164	4.6	491	13.7	38	1.1	964	26.8

Difference between positive results in females and males, assessed by Student’s t-test: *P* = 0.432, difference not significant

Variation depending on age, assessed by χ^2^ test: χ^2^ = 18.109, *P* = 0.006, variation significant

Prevalence of the anti-*B. burgdorferi* antibodies of the IgG class (14.7 %) was similar to that of the IgM class (16.0 %), although the majority of seropositive reactions was different in both classes. In spite of this, a significant relationship was found between the total (positive and negative) reactions in both classes of antibodies (χ^2^ = 52.80; *P* < 0.00001).

Positive and statistically significant relationships were found between a seropositive reaction to *B. burgdorferi* and the following questionnaire data: living in the country for more than 10 years (*P* < 0.01), living close to forested area (*P* < 0.0001), frequent presence in the forest (*P* < 0.0001), spending time in the forest or its vicinity for more than 6 h daily (*P* < 0.05), experienced tick bite (*P* < 0.0001), performed tests for borreliosis (*P* < 0.01), positive result of the test for borreliosis (*P* < 0.0001), diagnosed or suspected borreliosis (*P* < 0.0001) (Table 3). Interesting associations were found between a seropositive reaction and reported frequency of tick bite: in persons reporting no bite, the prevalence of reactions was significantly smaller (*P* < 0.0001), in those reporting one bite, there was no significant association in any direction (*P* = 0.378), while in those reporting 2–10 and 11–20 bites, the prevalence of reactions was significantly greater (*P* < 0.05 and *P* < 0.0001, respectively) (Table 3).

**Table 3:** Associations between questionnaire data and serological reactions to *B. burgdorferi*

Question	Seropositive to *B. burgdorferi* ^a^	Seronegative to *B. burgdorferi*	Odds ratio (OR)	95 % CI	P_OR_	Significance
1. Duration of living in country						
<2 years	15/880 (1.7 %)	73/2414 (3.0 %)	0.556	0.317–0.975	*P* = 0.040	+
2–5 years	8/880 (0.9 %)	19/2414 (0.8 %)	1.156	0.504–2.651	*P* = 0.731	NS
6–10 years	7/880 (0.8 %)	59/2414 (2.5 %)	0.320	0.146–0.703	*P* = 0.0046	++
>10 years	850/880 (96.6 %)	2263/2414 (93.7 %)	1.891	1.268–2.819	*P* = 0.0018	++
2. Living close to wooded area						
Yes	659/923 (71.4 %)	1624/2534 (64.1 %)	1.399	1.187–1.648	*P* < 0.0001	+++
No	264/923 (28.6 %)	910/2534 (35.9 %)	0.715	0.607–0.843	*P* = 0.0001	+++
3. Frequent presence in the forest						
Yes	311/922 (33.7 %)	673/2548 (26.4 %)	1.418	1.205–1.668	*P* < 0.0001	+++
No	164/922 (17.8 %)	553/2548 (21.7 %)	0.780	0.643–0.947	*P* = 0.012	+
Accidental	447/922 (48.5 %)	1322/2548 (51.9 %)	0.873	0.751–1.015	*P* = 0.077	NS
4. Number of hours spent daily in the forest or in its vicinity						
1–3 h	437/636 (68.7 %)	1177/1612 (73.0 %)	0.812	0.664–0.992	*P* = 0.0413	+
4–6 h	58/636 (9.1 %)	137/1612 (8.5 %)	1.080	0.783–1.490	*P* = 0.638	NS
>6 h	141/636 (22.2 %)	298/1612 (18.5 %)	1.256	1.003–1.574	*P* = 0.0475	+
5. Experienced a tick bite						
Yes	497/923 (53.9 %)	1079/2554 (42.3 %)	1.595	1.371–1856	*P* < 0.0001	+++
No	290/923 (31.4 %)	1076/2554 (42.1 %)	0.629	0.536–0.738	*P* < 0.0001	+++
Does not remember	136/923 (14.7 %)	399/2554 (15.6 %)	NC	NC	NC	NC
6. Frequency of tick bite						
Never	146/758 (19.3 %)	620/2007 (30.9 %)	0.534	0.435–0.654	*P* < 0.0001	+++
Once	116/758 (15.3 %)	335/2007 (16.7 %)	0.902	0.717–1.349	*P* = 0.378	NS
2–10	240/758 (31.6 %)	537/2007 (26.8 %)	1.268	1.057–1.522	*P* = 0.011	+
11–20	78/758 (10.3 %)	99/2007 (4.9 %)	2.211	1.622–3.812	*P* < 0.0001	+++
Does not remember	178/758 (23.5 %)	416/2007 (20.7 %)	NC	NC	NC	NC
7. Performed tests for borreliosis						
Yes	135/909 (14.9 %)	281/2515 (11.2 %)	1.387	1.112–1.729	*P* = 0.0037	++
No	719/909 (79.1 %)	2032/2515 (80.8 %)	0.899	0.745–1.086	*P* = 0.270	NS
Does not remember	55/909 (6.0 %)	202/2515 (8.0 %)	NC	NC	NC	NC
8. Result of the test for borreliosis^b^						
Positive	62/158 (39.2 %)	41/372 (11.0 %)	5.214	3.307–8.220	*P* < 0.0001	+++
Negative	43/158 (27.2 %)	171/372 (46.0 %)	0.439	0.293–0.659	*P* = 0.0001	+++
Does not remember	53/158 (33.6 %)	160/372 (43.0 %)	NC	NC	NC	NC
9. Diagnosed or suspected borreliosis						
Yes	85/837 (10.2 %)	81/2286 (3.5 %)	3.071	2.241–4.210	*P* < 0.0001	+++
No	690/837 (82.4 %)	2003/2286 (87.6 %	0.663	0.534–0.824	*P* = 0.0002	+++
Does not remember	63/837 (7.4 %)	201/2286 (8.9 %)	NC	NC	NC	NC
10. Inspection of the body after return from the forest						
Yes	590/896 (65.8 %)	1536/2453 (62.6 %)	1.151	0.980–1.352	*P* = 0.086	NS
No	135/896 (15.1 %)	450/2453 (18.3 %)	0.790	0.640–0.974	*P* = 0.027	+
Rarely	171/896 (19.1 %)	469/2453 (19.1 %)	1.050	0.863–1.276	*P* = 0.626	NS
11. Removal of ticks from body^c^						
By self	329/580 (56.7 %)	708/1355 (52.3 %)	1.198	0.985–1.457	*P* = 0.071	NS
By doctor or nurse	97/580 (16.7 %)	298/1355 (22.0 %)	0.712	0.553–0.917	*P* = 0.0086	++
With help of other persons	172/580 (29.7 %)	358/1355 (26.4 %)	1.174	0.947–1.456	*P* = 0.144	NS
12. Mode of tick removal^c^						
With tweezers	302/555 (54.4 %)	654/1206 (54.2 %)	1.007	0.823–1.233	*P* = 0.942	NS
With specially designed commercial instruments	30/555 (5.4 %)	59/1206 (4.9 %)	1.111	0.707–1.745	*P* = 0.648	NS
With fingers	228/555 (41.1 %)	472/1206 (39.1 %)	1.084	0.883–1.331	*P* = 0.439	NS
By other mode	34/555 (6.1 %)	89/1206 (7.4 %)	0.819	0.544–1.232	*P* = 0.338	NS
13. Use of repellents						
Yes	152/873 (17.4 %)	448/2373 (18.9 %)	0.906	0.739–1.110	*P* = 0.340	NS
No	540/873 (61.9 %)	1496/2373 (63.0 %)	0.951	0.810–1.116	*P* = 0.535	NS
Accidental	181/873 (20.7 %)	429/2373 (18.1 %)	1.185	0.976–1.439	*P* = 0.086	NS

Explanation: In the fields “Seropositive to *B. burgdorferi*” and “Seronegative to *B. burgdorferi*” are given: Total positive to particular question/total examined (in parentheses: percent of seropositive or seronegative respondents to this question)

*OR* odds ratio, *95 % CI* 95 % confidence interval, *P*_OR_ probability calculated for OR

Significance: *NS* = not significant; +++ = *P* < 0.001; ++ = *P* < 0.01; + = *P* < 0.05

*NC* = not calculated

^a^ Including borderline results

^b^Persons who answered “Yes” or “Not remember” to question # 7 were tested

^c^Some respondents indicated 2–4 answers

The relationships between a seropositive reaction to *B. burgdorferi* and the prevention measures applied by the investigated farmers were mostly not significant and inconclusive, except for an association between the skilful removal of ticks by doctor and nurse and decrease of seropositive reactions. However, this dependency could be accidental, similar to an association between lack of body inspection after return from the forest and decrease in seropositive reactions (Table 3).

## Discussion

The diversity of *B. burgdorferi* strains creates tremendous difficulties in the development the universal diagnostic tests. The heterogeneity of antigens (native or recombinant proteins) and differences in methods of antibodies detection used by laboratories lead to the problems associated with comparison of variable results. Immunoenzymatic methods, such as ELISA using genetic recombinants as antigen (III generation of tests), increase sensitivity and specificity and could be successfully used for screening studies of borreliosis [15]. According to European guidelines about two-step diagnostics of borreliosis, all positive results in ELISA test should be confirmed by Western blot. In this study, only a screening method (ELISA) was used and the positive results were not confirmed by Western blot. Our previous study using the same serological kits showed that all the positive and borderline sera in ELISA were confirmed as positive also with Western blot test [16]. Nevertheless, this result does not preclude the reliability of Western blot which is a good confirmatory test in the serodiagnostics of Lyme borreliosis, allowing for exclusion false-positive reactions. In this study, the Western blot test has not been used because of financial limitations, but all the farmers with positive or borderline ELISA result were advised to consult a medical practitioner and perform the Western blot test.

Accordingly, in evaluation of the results of this study, some limitations of the ELISA test must be considered. The test may give false-positive cross-reactions with some agents of other infectious diseases, such as Epstein-Barr virus (EBV), *Helicobacter pylori* or *Treponema pallidum* or in the presence of autoantibodies, such as a rheumatoid factor. On the other side, false-negative reactions may occur in the presence of immunosuppression, during antibiotic treatment or after a long time that elapsed since clinically diagnosed Lyme borreliosis. As in the questionnaire survey performed in the present study only two of the examined farmers reported the presence of existing autoimmune disease (both patients had negative results), and another two reported infectious diseases and applied antibiotic treatment (one of them tested positive and one tested negative); the common occurrence of false-positive reactions due to infectious or autoimmune diseases or false-negative reactions due to antibiotic application seems not very probable. Nevertheless, the presence of such reactions cannot be excluded, as with the mild symptoms farmers could not seek medical help and some disease cases might not be properly diagnosed. More probable in the group of farmers that we examined could be false-negative reactions in the individuals with clinical Lyme borreliosis diagnosed in the past, as in 166 individuals with such diagnosis only 85 (51.2 %) showed positive or borderline reactions to *B. burgdorferi* antigen, mostly those associated with IgG class (58 farmers). Unfortunately, the data on the exact time that elapsed from the episode of clinical Lyme borreliosis were not available in all cases, so we assumed that in most cases it exceeded 3 years. Nevertheless, in farmers with the past Lyme borreliosis the occurrence of false-negative reactions with the presence of living encysted spirochetes cannot be excluded.

Our previous study [16], showed that 33.0 % of 94 examined farmers in the Lublin area had specific IgG and/or IgM antibodies against *B. burgdorferi*, which is a little higher in comparison with the present study results obtained on a much greater group—26.8 % of 3,597 farmers and similar to another previous study (27.3 %) [9]. By comparison, Cisak et al. [9] reported in the control group of urban blood donors only 7 % positive results.

Higher seroprevalence among farmers (42.3 %) from the neighbouring regions of South Podlasie Lowland and Lublin Polesie was reported by Tokarska-Rodak et al. [11] with the use of the ELISA method, of which only 28 % were confirmed by Western blot test. Our results in IgG class (14.8 %) were much lower compared to those obtained in France, where 25 % of farmers had specific antibodies detected by microimmunofluorescence [17].

In contrast, our results are much higher than those reported by Kaya et al. [18] from Turkey where only 10.9 % of studied farmers and forestry workers had specific antibodies, detected only in IgG class. Simultaneously, we observed the increase of positive reaction frequency in the oldest age ranges, while studies by Kaya et al. [18] showed inverse correlation. Similarly, the seroprevalence stated by us was about 2.5 times higher than in Italy (10 %) [19]. In Sweden, the prevalence of *B. burgdorferi* antibodies was only 7.6 % in farmers and forestry workers, and in this area there was probably no occupational risk among outdoor workers [20]. Lower seroprevalence was also reported by Angelov et al. [21] in Bulgaria and by Stanford et al. [12] in Ireland (17.8 and 14.3 %, respectively).

In this study, the seroprevalence varied between localities and ranged from 18.2 to 50.7 %, suggesting a focal occurrence of Lyme borreliosis. The prevalence of anti-*B. burgdorferi* antibodies in sera of farmers examined in the present study showed a correlation with the infection rates of ticks with *B. burgdorferi* estimated in previous studies [22], e.g. high seroprevalence rates in the districts of Włodawa (50.7 %) and Radzyń Podlaski and Parczew (32.5 %) were associated with higher infection rates of ticks from these areas (5.6 and 10.9 %, respectively), while lower seroprevalence rates stated in the districts of Kraśnik (22.7 %) and Zamość (24.2 %) were associated with lower infection rates of ticks (4.3 and 2.9 %, respectively). Our results indicating that Hajnówka is a region of high risk of Lyme borreliosis (seroprevalence equal to 35.9 %) correspond to the fact that in the year 2000, the closely situated region of the Białowieża Primeval Forest was characterized by a very high morbidity of Lyme disease (118.4/100,000), compared to the whole country (4.79/100,000) [23].

Farmers, as a professional group working in forests or meadows with surroundings being natural ecosystems for ticks, are occupationally exposed to high risk of tick-borne infections. Our studies showed a high prevalence of positive serologic reactions to the Spotted Fever Group (SFG) rickettsiae, tick-borne encephalitis virus (TBEV) and *Bartonella henselae* in the examined agricultural workers in Lublin province (21.3, 21.7 and 27.7 %, respectively) [8, 24, 25]. According to the Central Register of Occupational Diseases recorded by the Nofer Institute of Occupational Diseases in Łódź, during 2000–2014, infectious and parasitic diseases accounted for 62 % of all occupational diseases among Polish farmers. In this group, tick-borne diseases were the most frequent (93 %) and Lyme borreliosis was the most common infection (85.8 %). The rate of Lyme borreliosis incidence among occupational diseases in farmers has increased from 16.8 % in 2000 to 76.3 % in 2014 [5]. According to the records of KRUS, the number of one-time compensation due to Lyme borreliosis among farmers is still rising, from 50 cases in 2007 to 176 in 2013. The increasing incidence of Lyme borreliosis may be explained by the rising number of infected ticks. As indicated in our recent study, the infection rate of the main tick vector *Ixodes ricinus* with *B. burgdorferi* s. l. significantly increased from 6.0 % in the years 2008–2009 up to 15.3 % in the years 2013–2014. In addition, single and mixed infections have been confirmed in the tick population with considerable increasing tendency [26].

According to the annual bulletin ‘Infectious diseases and poisonings in Poland’, in a period of 10 years (2005–2015) [27], the incidence of Lyme borreliosis in Poland increased from 11.5 to 35.4 per 100,000 population. Increased incidence was observed also for particular voivodeships: Lubelskie (from 8.6–51), Masovian (3.2–26.8) and Podlaskie (63.4–96.3). Surprisingly, in the Masovian voivodeship with the lowest incidences of Lyme borreliosis, the highest seroprevalence (28.0 %) was found in this study. Podlaskie and Lubelskie voivodeships had very similar seroprevalence (26.2 % vs. 26.6 %); however, Lyme borreliosis incidence in Podlaskie was twice as high as in Lubelskie. This fact can be explained by the presence of serological response to *Borrelia* antigen in people frequently exposed to tick bite who, however, do not develop clinical symptoms. As indicated by Cinco et al. [28], positive serology in the absence of clinical symptoms could be a result of repeated exposures to *B. burgdorferi*, which consequently could lead to natural re-vaccination and acquired immunity to Lyme borreliosis.

In our study, seroreactivity to *B. burgdorferi* antigen was higher in the group of farmers exposed to repeated tick bites, compared to the study by Kaya et al. [18] who did not observe such dependence. Our results indicate that one tick bite may be not associated with the infection manifested by seropositive reaction, whereas multiple bites may significantly increase the chance for infection and seroconversion. Different results were obtained by Zákutná et al. [29] in Slovakia who reported that 15 % of healthy blood donors showed the presence of positive serologic reactions, but association between seroprevalence and spending time at a cottage was not observed. By contrast, in our study, clearly significant relationships were found between seropositive reaction to *B. burgdorferi* and such risk factors as living in the country or near a forested area, and frequent presence in the forest, which was also reported by Cinco et al. [28]. Conversely, the relationships between a seropositive reaction to *B. burgdorferi* and the prevention measures applied by the investigated farmers appeared mostly as not significant and inconclusive. Thus, the important subject of the best protection for farmers against Lyme borreliosis and other tick-borne diseases should be a subject of future studies.

## Conclusions

To the best of our knowledge, this is the first report on *B. burgdorferi s*eroprevalence carried out on such a large group of farmers (3,597 subjects). The results indicate a high risk of *B. burgdorferi* infection among farmers in various regions of eastern and central Poland. We confirmed associations between seroprevalence and a range of risk factors. Further screening studies on occupational risk groups are recommended for assurance of a better protection of individuals exposed to tick bite.
